# Glucagon-Like Peptide-1 Receptor Agonist Cases Reported to United States Poison Centers, 2017–2022

**DOI:** 10.1007/s13181-024-00999-x

**Published:** 2024-02-29

**Authors:** Christopher E. Gaw, Hannah L. Hays, Cydney A. Kemp, Sandhya Kistamgari, Henry A. Spiller, Natalie I. Rine, Allison L. Rhodes, Motao Zhu, Gary A. Smith

**Affiliations:** 1 Center for Injury Research and Policy, The Abigail Wexner Research Institute at Nationwide Children’s Hospital, 700 Children’s Drive, 43205 Columbus, OH USA; 2 Division of Emergency Medicine, Nationwide Children’s Hospital, Columbus, OH USA; 3 Department of Pediatrics, The Ohio State University College of Medicine, Columbus, OH USA; 4 Section of Toxicology, Nationwide Children’s Hospital, Columbus, OH USA; 5 Central Ohio Poison Center, Nationwide Children’s Hospital, Columbus, OH USA; 6 Campbell University School of Osteopathic Medicine, Lillington, NC USA; 7 Division of General Internal Medicine, The Ohio State University College of Medicine, Columbus, OH USA; 8 Child Injury Prevention Alliance, Columbus, OH USA

**Keywords:** Adverse drug events, GLP-1 receptor agonist, Semaglutide, Liraglutide, Toxicity

## Abstract

**Introduction:**

Glucagon-like peptide-1 receptor agonists (GLP-1RAs) are a class of medications for management of diabetes and obesity. The objective of this study is to characterize the epidemiology of GLP-1RA cases reported to US poison centers.

**Methods:**

We analyzed cases involving a GLP-1RA reported to the National Poison Data System during 2017–2022.

**Results:**

There were 5,713 single-substance exposure cases reported to US poison centers involving a GLP-1RA. Most cases were among females (71.3%) and attributable to therapeutic errors (79.9%). More than one-fifth (22.4%) of cases were evaluated in a healthcare facility, including 0.9% admitted to a critical care unit and 4.1% admitted to a non-critical care unit. Serious medical outcomes were described in 6.2% of cases, including one fatality. The rate of cases per one million US population increased from 1.16 in 2017 to 3.49 in 2021, followed by a rapid increase of 80.9% to 6.32 in 2022. Trends for rates of serious medical outcomes and admissions to a healthcare facility showed similar patterns with 129.9% and 95.8% increases, respectively, from 2021 to 2022.

**Conclusions:**

Most GLP-1RA cases reported to US poison centers were associated with no or minimal effects and did not require referral for medical treatment; however, a notable minority of individuals experienced a serious medical outcome or healthcare facility admission. The rate of reported cases increased during the study period, including an 80.9% increase from 2021 to 2022. Opportunities exist to improve provider and patient awareness of the adverse effects of these medications.

**Supplementary Information:**

The online version contains supplementary material available at 10.1007/s13181-024-00999-x.

## Introduction

Glucagon-like peptide-1 receptor agonists (GLP-1RAs) are a class of medications commonly used in the treatment of type II diabetes mellitus and, more recently, obesity [1, 2]. Although only three GLP-1RAs — Saxenda (liraglutide), Wegovy (semaglutide), and Zepbound (tirzepatide, which is also a glucose-dependent insulinotropic polypeptide receptor agonist) — are approved in the United States (US) for chronic weight management in adults by the US Food and Drug Administration (FDA) [3–5], the medication class has become a popular off-label option for weight loss [6]. Provider awareness combined with intense public interest has precipitated high levels of prescribing and subsequent drug shortages [7]. Evidence that the use of GLP-1RAs improves outcome for heart failure and nonalcoholic steatohepatitis has been published, which could further increase off-label usage [8, 9]. Globally valued at $22.4 billion in 2022, the market size of GLP-1RA medications is expected to demonstrate an annual growth rate of approximately 9.6% through 2032 [10].

GLP-1RAs demonstrate substantial homology with endogenous GLP-1 but have been structurally modified to resist rapid degradation by dipeptidyl peptidase-4 [1, 11]. These modifications vary slightly among drugs in this class, but generally result in enhanced binding to albumin, which slows renal elimination of the drug [11]. Thus, GLP-1RAs are slowly degraded, have relatively long half-lives, and can exert an array of potent effects, including increased satiety, enhanced insulin secretion, and suppression of glucagon [2, 11, 12]. Although these physiologic effects are leveraged for glucose control, beta-cell rehabilitation, and weight loss, adverse effects can also occur. Gastrointestinal side effects, such as nausea, vomiting, and constipation, are consistently described in clinical trials, systematic reviews, and case reports [13–18]. GLP-1RA use has also been associated with cases of pancreatitis, biliary disease, gastroparesis, bowel obstruction, and hypersensitivity reactions [14, 19–23].

Most of the adverse effects related to GLP-1RAs are described in clinical trial settings [13, 15, 16]. With the increased prescribing of this medication class for both on- and off-label indications, continued population-level studies on adverse effects related to these medications are critical. US poison center (PC) data provide a unique opportunity to complement post-marketing drug surveillance data to describe potential adverse events related to GLP-1RA use. In this study, we characterize GLP-1RA cases reported to US PCs using data from the National Poison Data System (NPDS).

## Methods

### Data Sources

We obtained data from the NPDS, a surveillance system maintained by America’s Poison Centers [24]. The NPDS captures information from contacts by the public and healthcare professionals to regional PCs that serve the entire US and all US territories. Trained Specialists in Poison Information who staff the 24-hour Poison Help Line record information in the NPDS as part of the routine triage and management of these cases. The NPDS employs quality control measures to promote the integrity and completeness of its data [24–26]. July 1st intercensal and postcensal population estimates were obtained from the US Census Bureau to calculate annual case rates [27, 28]. This study was determined to be exempt from review by the institutional review board at the authors’ institution.

### Case Selection Criteria

Single-substance cases involving a GLP-1RA reported to US PCs between January 1, 2017 and December 31, 2022 were identified based on NPDS generic and product codes. Cases were excluded if the medical outcome was documented as a “confirmed non-exposure” (*n* = 246 cases) or “unrelated effect” (*n* = 24 cases), which left 5,713 cases for analysis.

### Study Variables

We grouped age into the following categories: 1) < 20 years (children/teenagers), 2) 20–49 years (adults), 3) 50–59 years (middle-aged adults), 4) 60–69 years (older adults #1), 5) > 69 years (older adults #2), and 6) unknown. Based on NPDS categories, the level of health care received was classified as: (1) no healthcare facility (HCF) treatment received, (2) treated/evaluated and released, (3) admitted to a critical care unit, (4) admitted to a non-critical care unit, (5) patient refused referral/did not arrive at a HCF, or (6) patient lost to follow-up/left against medical advice. In this study, “patients lost to follow-up/left against medical advice” were treated as “unknown” in analyses. Based on NPDS product codes, substances were categorized into three groups: (1) semaglutide, (2) liraglutide, and (3) other GLP-1RAs. Semaglutide and liraglutide were grouped separately from other GLP-1RAs because, at the time of the study, they were the only two with a FDA-approved indication for chronic weight management (tirzepatide was not approved for this purpose until November 2023) [3, 4].

Medical outcomes, as defined by the NPDS [26], were classified as follows: (1) no effect, (2) minor effect (minimal symptoms that generally resolve rapidly), (3) moderate effect (more pronounced, prolonged, or systemic symptoms than minor effect), (4) major effect (symptoms that are life-threatening or result in substantial disability or disfigurement), (5) death, (6) not followed (minimal clinical effects possible), or (7) unable to follow (judged as potentially toxic exposure). Cases that were “unable to follow (judged as potential toxic exposure)” were treated as “unknown” in analyses. In this study, the category “serious medical outcome” combined the outcomes reported as a moderate effect, major effect, or death. Additional variables examined included sex, year, exposure type (single-substance or poly-substance), reason for exposure, therapeutic error scenario, and related clinical effects.

### Statistical Analysis

Data were analyzed using SPSS Statistics 28.0 (IBM Corporation, Armonk, NY) and SAS 9.4 (SAS Institute, Inc. Cary, NC). Descriptive statistics and rates were reported. Odds ratios (ORs) with corresponding 95% confidence intervals (CIs) were calculated to determine the magnitude of association between product category and HCF admission and between product category and serious medical outcome.

## Results

### General Characteristics

Among the 5,713 cases reported to US PCs involving a GLP-1RA as single substance, 71.3% involved females and 98.4% occurred at home (Table 1). Although some cases had more than one route of exposure, 76.3% of cases were associated with injection, and 17.0% with ingestion. Age distribution was individuals < 20 years old (5.8%), 20–49 years old (29.3%), 50–59 years old (27.0%), 60–69 years old (23.8%), and > 69 years old (14.0%).

**Table 1:** Characteristics of cases involving a GLP-1 receptor agonist reported to the NPDS by age group, 2017–2022

Characteristic	Age Group						
	< 20 Years	20–49 Years	50–59 Years	60–69 Years	> 69 Years	Unknown	Total
	n (%) ^a^	n (%) ^a^	n (%) ^a^	n (%) ^a^	n (%) ^a^	n	n (%) ^a^
**Product Category**							
Semaglutide	86 (28.9)	746 (49.5)	599 (43.1)	503 (41.0)	241 (33.4)	250	2,425 (42.5)
Liraglutide	44 (14.8)	357 (23.7)	329 (23.7)	300 (24.5)	180 (25.0)	131	1,341 (23.5)
Other GLP-1 Receptor Agonists	168 (56.4)	404 (26.8)	462 (33.2)	423 (34.5)	300 (41.6)	190	1,947 (34.1)
**Sex**							
Male	149 (50.0)	348 (23.2)	355 (25.6)	365 (29.8)	259 (35.9)	159	1,635 (28.7)
Female	149 (50.0)	1,155 (76.9)	1,033 (74.4)	861 (70.2)	462 (64.1)	400	4,060 (71.3)
Unknown	0	4	2	0	0	12	18
**Reason for Exposure**							
Unintentional	265 (90.1)	1,264 (84.0)	1,273 (91.7)	1,123 (91.7)	680 (94.8)	491	5,096 (89.5)
Unintentional– General	199 (67.7)	59 (3.9)	55 (4.0)	49 (4.0)	29 (4.0)	36	427 (7.5)
Unintentional– Therapeutic error	53 (18.0)	1,173 (77.9)	1,195 (86.1)	1,045 (85.4)	641 (89.4)	444	4,551 (79.9)
Unintentional– Other	13 (4.4)	32 (2.1)	22 (1.6)	28 (2.3)	10 (1.4)	11	116 (2.0)
Unintentional - Unknown	0 (0.0)	0 (0.0)	1 (0.1)	1 (0.1)	0 (0.0)	0	2 (0.0)
Intentional	25 (8.5)	104 (6.9)	36 (2.6)	28 (2.3)	6 (0.8)	20	219 (3.8)
Intentional– Suspected suicide	14 (4.8)	48 (3.2)	12 (0.9)	9 (0.7)	3 (0.4)	5	91 (1.6)
Intentional– Misuse	8 (2.7)	51 (3.4)	21 (1.5)	14 (1.1)	3 (0.4)	13	110 (1.9)
Intentional - Abuse	0 (0.0)	1 (0.1)	3 (0.2)	1 (0.1)	0 (0.0)	0	5 (0.1)
Intentional - Unknown	3 (1.0)	4 (0.3)	0 (0.0)	4 (0.3)	0 (0.0)	2	13 (0.2)
Other	4 (1.4)	137 (9.1)	79 (5.7)	73 (6.0)	31 (4.3)	58	382 (6.7)
Unknown	4	2	2	2	4	2	16
**Highest Level of Health Care Received**							
No health care facility treatment referral	134 (48.7)	941 (66.9)	1,021 (76.8)	912 (78.1)	547 (79.2)	480	4,035 (74.9)
Treated/evaluated and released	91 (33.1)	326 (23.2)	217 (16.3)	167 (14.3)	102 (14.8)	18	921 (17.1)
Admitted to a health care facility	39 (14.2)	91 (6.5)	54 (4.1)	62 (5.3)	39 (5.6)	3	288 (5.3)
Admitted to critical care unit	7 (2.6)	20 (1.4)	6 (0.5)	7 (0.6)	6 (0.9)	0	46 (0.9)
Admitted to non-critical care unit	27 (9.8)	59 (4.2)	47 (3.5)	53 (4.5)	32 (4.6)	3	221 (4.1)
Admitted to psychiatric facility	5 (1.8)	12 (0.9)	1 (0.1)	2 (0.2)	1 (0.1)	0	21 (0.4)
Refused referral/did not arrive at health care facility	11 (4.0)	49 (3.5)	37 (2.8)	27 (2.3)	3 (0.4)	17	144 (2.7)
Unknown ^b^	23	100	61	58	30	53	325
**Medical Outcome**							
No effect	101 (35.7)	229 (16.2)	258 (19.4)	260 (22.3)	194 (27.7)	65	1,107 (20.5)
Minor effect	60 (21.2)	354 (25.0)	220 (16.5)	187 (16.1)	79 (11.3)	42	942 (17.4)
Serious medical outcome	13 (5.5)	119 (8.4)	96 (7.3)	59 (5.1)	33 (4.7)	18	338 (6.2)
Moderate effect	12 (4.2)	112 (7.9)	94 (7.1)	56 (4.8)	29 (4.1)	17	320 (5.9)
Major effect	1 (0.4)	6 (0.4)	2 (0.2)	3 (0.3)	4 (0.6)	1	17 (0.3)
Death	0 (0.0)	1 (0.1)	0 (0.0)	0 (0.0)	0 (0.0)	0	1 (0.0)
Not followed ^c^	109 (38.5)	713 (50.4)	757 (56.9)	658 (56.5)	394 (56.3)	382	3,013 (55.8)
Unknown ^d^	15	92	59	62	21	64	313
**Total (row %)** ^e^	**298 (5.8)**	**1,507 (29.3)**	**1,390 (27.0)**	**1,226 (23.8)**	**721 (14.0)**	**571**	**5,713 (100.0)**

^a^ Column percentages may not add to 100.0% due to rounding error

^b^ Includes “patient lost to follow-up/ left against medical advice” and cases for which management site was recorded as “unknown”

^c^ Includes “not followed (judged as a non-toxic exposure)” and “not followed (minimal clinical effects possible)”

^d^ Includes “unable to follow (judged as a potentially toxic exposure)”

^e^ Row percentages may not sum to 100.0% because of rounding error

Most (79.9%) reported cases were associated with therapeutic errors, while 3.8% were intentional in nature. Among the 4,551 cases associated with a therapeutic error, the most common scenario was “inadvertently took/given medication twice” (29.8%), followed by “other incorrect dose” (21.9%) and “medication doses taken/given too close together” (17.7%). Among the 219 intentional cases, 41.6% were coded as “intentional - suspected suicides” and 52.5% were attributed to misuse/abuse. Most “intentional - suspected suicides” were among the 20-49-year-old age group (52.7%), followed by < 20 years old (15.4%,) and 50-59-year-olds (13.2%). Most misuse/abuse cases were among 20-49-year-olds (45.2%), followed by individuals 50–59 years old (20.9%) and 60–69 years old (13.0%).

### Highest Level of Health Care Received and Medical Outcomes

Most cases were not referred to a HCF for treatment (74.9%) or refused referral/did not arrive at a HCF (2.7%) (Table 1). Of the 22.4% (*n* = 1,209) of cases evaluated in a HCF, 17.1% were treated/evaluated and released, 0.9% admitted to a critical care unit, 4.1% admitted to a non-critical care unit, and 0.4% admitted to a psychiatric facility.

More than half (55.8%) of cases were not followed because they were judged to have a non-toxic exposure or a possibility of minimal clinical effects. An additional 20.5% had no effects and 17.4% had minor effects. Serious medical outcomes were identified in 6.2% of cases, including 17 cases (0.3%) with major effects. There was one fatality of a 39-year-old female reported to the NPDS involving a GLP-1RA in the “other GLP-1RA” category. It was coded as a single-substance unintentional adverse drug reaction. Case notes describe the patient presenting for care after developing acute onset of abdominal pain and distension in the setting of constipation. Imaging and exploratory laparotomy were performed and revealed colonic distension, fecal impaction, and proctocolitis with subsequent bowel ischemia. The fatality occurred following multiple operations and a multi-day critical care course complicated by bowel ischemia and resection, suspected gastrointestinal bleeding, and 2 episodes of cardiac arrest (the latter with failure to achieve return of spontaneous circulation). The case was reported to the FDA MedWatch system as a possible adverse event related to the GLP-1RA.

### Product Category

The most common product category was semaglutide (42.5%), followed by other types of GLP-1RAs (34.1%) and liraglutide (23.5%) (Table 2). Among semaglutide cases reported to US PCs, 5.6% were admitted to a HCF, compared with 4.7% for liraglutide and 5.5% for the other GLP-1RAs combined. In addition, 7.7% of semaglutide cases were associated with serious medical outcomes compared with 4.7% for liraglutide cases and 5.6% for the other GLP-1RA cases combined. Compared with all non-semaglutide-related cases combined, semaglutide cases did not have significantly different odds of admission to a HCF (OR: 1.04, 95% CI: 0.82–1.32) but did have significantly greater odds of a serious medical outcome (OR: 1.45, 95% CI: 1.17–1.81).

**Table 2:** Characteristics of cases involving a GLP-1 receptor agonist as the primary substance reported to the NPDS by product group, 2017–2022

Characteristic	Product Category			
	Semaglutide	Liraglutide	Others	Total
	n (%) ^a^	n (%) ^a^	n (%) ^a^	n (%) ^a^
Sex				
Male	713 (29.5)	321 (24.0)	601 (31.0)	1,635 (28.7)
Female	1,704 (70.5)	1,016 (76.0)	1,340 (69.0)	4,060 (71.3)
Unknown	8	4	6	18
Age Group (Years)				
< 20	86 (4.0)	44 (3.6)	168 (9.6)	298 (5.8)
20–49	746 (34.3)	357 (29.5)	404 (23.0)	1,507 (29.3)
50–59	599 (27.5)	329 (27.2)	462 (26.3)	1,390 (27.0)
60–69	503 (23.1)	300 (24.8)	423 (24.1)	1,226 (23.8)
> 69	253 (11.6)	180 (14.9)	300 (17.1)	721 (14.0)
Unknown	250	131	190	571
Reason for Exposure				
Unintentional	2,135 (88.3)	1,211 (90.6)	1,750 (90.1)	5,096 (89.5)
Unintentional– General	127 (5.3)	56 (4.2)	244 (12.6)	427 (7.5)
Unintentional– Therapeutic error	1,991 (82.3)	1,144 (85.6)	1,416 (72.9)	4,551 (79.9)
Unintentional– Other	17 (0.7)	9 (0.7)	90 (4.6)	116 (2.0)
Unintentional - Unknown	0 (0.0)	2 (0.1)	0 (0.0)	2 (0.0)
Intentional	89 (3.7)	65 (4.9)	65 (3.3)	219 (3.8)
Intentional– Suspected suicide	28 (1.2)	35 (2.6)	28 (1.4)	91 (1.6)
Intentional– Misuse	54 (2.2)	25 (1.9)	31 (1.6)	110 (1.9)
Intentional - Abuse	3 (0.1)	1 (0.1)	1 (0.1)	5 (0.1)
Intentional - Unknown	4 (0.2)	4 (0.3)	5 (0.3)	13 (0.2)
Other	195 (8.1)	60 (4.5)	127 (6.5)	382 (6.7)
Unknown	6	5	5	16
Highest Level of Health Care Received				
No health care facility treatment referral	1,577 (70.1)	1,013 (79.1)	1,445 (77.8)	4,035 (74.9)
Treated/evaluated and released	473 (21.0)	177 (13.8)	271 (14.6)	921 (17.1)
Admitted	125 (5.6)	60 (4.7)	103 (5.5)	288 (5.3)
Admitted to critical care unit	18 (0.8)	7 (0.6)	21 (1.1)	46 (0.9)
Admitted to non-critical care unit	103 (4.6)	45 (3.5)	73 (3.9)	221 (4.1)
Admitted to psychiatric facility	4 (0.2)	8 (0.6)	9 (0.5)	21 (0.4)
Refused referral/did not arrive at health care facility	76 (3.4)	30 (2.3)	38 (2.1)	144 (2.7)
Unknown ^b^	174	61	90	325
Medical Outcome				
No effect	378 (16.8)	284 (22.2)	445 (23.8)	1,107 (20.5)
Minor effect	472 (20.9)	188 (14.7)	282 (15.1)	942 (17.4)
Serious medical outcome	173 (7.7)	60 (4.7)	105 (5.6)	338 (6.3)
Moderate effect	165 (7.3)	56 (4.4)	99 (5.3)	320 (5.9)
Major effect	8 (0.4)	4 (0.3)	5 (0.3)	17 (0.3)
Death	0 (0.0)	0 (0.0)	1 (0.1)	1 (0.0)
Not followed ^c^	1,233 (54.7)	745 (58.3)	1,035 (55.4)	3,013 (55.8)
Unknown ^d^	169	64	80	313
Total (row %) ^e^	2,425 (42.5)	1,341 (23.5)	1,947 (34.1)	5,713 (100.0)

^a^ Column percentages may not add to 100.0% due to rounding error

^b^ Includes “patient lost to follow-up/ left against medical advice” and cases for which management site was recorded as “unknown”

^c^ Includes “not followed (judged as a non-toxic exposure)” and “not followed (minimal clinical effects possible)”

^d^ Includes “unable to follow (judged as a potentially toxic exposure)”

^e^ Row percentages may not add to 100.0% due to rounding error

### Related Clinical Effects

The most commonly reported related clinical effects included nausea (17.4%, *n* = 993), vomiting (13.9%, *n* = 791), diarrhea (3.4%, *n* = 192), and abdominal pain (3.3%, *n* = 190) (Table 3). Among cases admitted to a critical care unit or non-critical care unit, the most common related clinical effects were nausea (34.5%, *n* = 92), vomiting (30.7%, *n* = 82), hypoglycemia (22.1%, *n* = 59), abdominal pain (9.7%, *n* = 26), and dizziness/vertigo (7.1%, *n* = 19), and additional selected related clinical effects included increased creatinine (1.9%, *n* = 5), pancreatitis (0.4%, *n* = 1), AST, ALT > 1,000 (0.4%, *n* = 1), renal failure (0.4%, *n* = 1), and ileus (0.4%, *n* = 1).

**Table 3:** Selected related clinical effects associated with GLP-1 receptor agonist cases reported to the NPDS by product group, 2017–2022

Related Clinical Effect ^a^	Product Category			
	Semaglutide	Liraglutide	Others	Total
	n (%) ^b^	n (%) ^b^	n (%) ^b^	n (%) ^b^
Nausea	596 (24.6)	229 (17.1)	168 (8.6)	993 (17.4)
Vomiting	503 (20.7)	191 (14.2)	97 (5.0)	791 (13.9)
Diarrhea	125 (5.2)	21 (1.6)	46 (2.4)	192 (3.4)
Abdominal pain	114 (4.7)	26 (1.9)	50 (2.6)	190 (3.3)
Dizziness/vertigo	102 (4.2)	32 (2.4)	32 (1.6)	166 (2.9)
Headache	78 (3.2)	32 (2.4)	31 (1.6)	141 (2.5)
Hypoglycemia	51 (2.1)	25 (1.9)	63 (3.2)	139 (2.4)
Other - miscellaneous	37 (1.5)	25 (1.9)	30 (1.5)	92 (1.6)
Dermal - irritation/pain	8 (0.3)	4 (0.3)	56 (2.9)	68 (1.2)
Other - gastrointestinal	40 (1.7)	6 (0.5)	11 (0.6)	57 (1.0)
Edema	5 (0.2)	2 (0.2)	45 (2.3)	52 (0.9)
Other - neurological	31 (1.3)	5 (0.4)	15 (0.8)	51 (0.9)
Central nervous system depression (mild)	35 (1.4)	4 (0.3)	11 (0.6)	50 (0.9)
Tachycardia	28 (1.2)	10 (0.8)	7 (0.4)	45 (0.8)
Diaphoresis	21 (0.9)	7 (0.5)	12 (0.6)	40 (0.7)
Anorexia	22 (0.9)	7 (0.5)	10 (0.5)	39 (0.7)
Muscle weakness	20 (0.8)	5 (0.4)	8 (0.4)	33 (0.6)
Erythema/flushed	9 (0.4)	5 (0.4)	16 (0.8)	30 (0.5)
Ocular - irritation/pain	3 (0.1)	3 (0.2)	22 (1.1)	28 (0.5)
Tremor	14 (0.6)	3 (0.2)	10 (0.5)	27 (0.5)
Agitation	10 (0.4)	0 (0.0)	6 (0.3)	16 (0.3)
Chest pain (including noncardiac)	8 (0.3)	2 (0.2)	5 (0.3)	15 (0.3)
Other - dermal	4 (0.2)	1 (0.1)	10 (0.5)	15 (0.3)
Pruritus	4 (0.2)	1 (0.1)	9 (0.5)	14 (0.3)
Confusion	8 (0.3)	2 (0.2)	4 (0.2)	14 (0.3)
**Total cases (row %)** ^**c**^	**2,425 (42.5)**	**1,341 (23.5)**	**1,947 (34.1)**	**5,713 (100.0)**

^a^ Related clinical effects were selected if documented in > 0.2% of total single-substance GLP-1 receptor agonist cases

^b^ Column percentages were calculated using the total number of single-substance cases by product group as the denominator. Percentages will not sum to 100.0% because (1) each exposure may result in 0, 1, or more clinical effects, and (2) not all clinical effects are listed in the table

^c^ Row percentages may not sum to 100.0% due to rounding error

### Trend Analysis

**Fig. 1:**
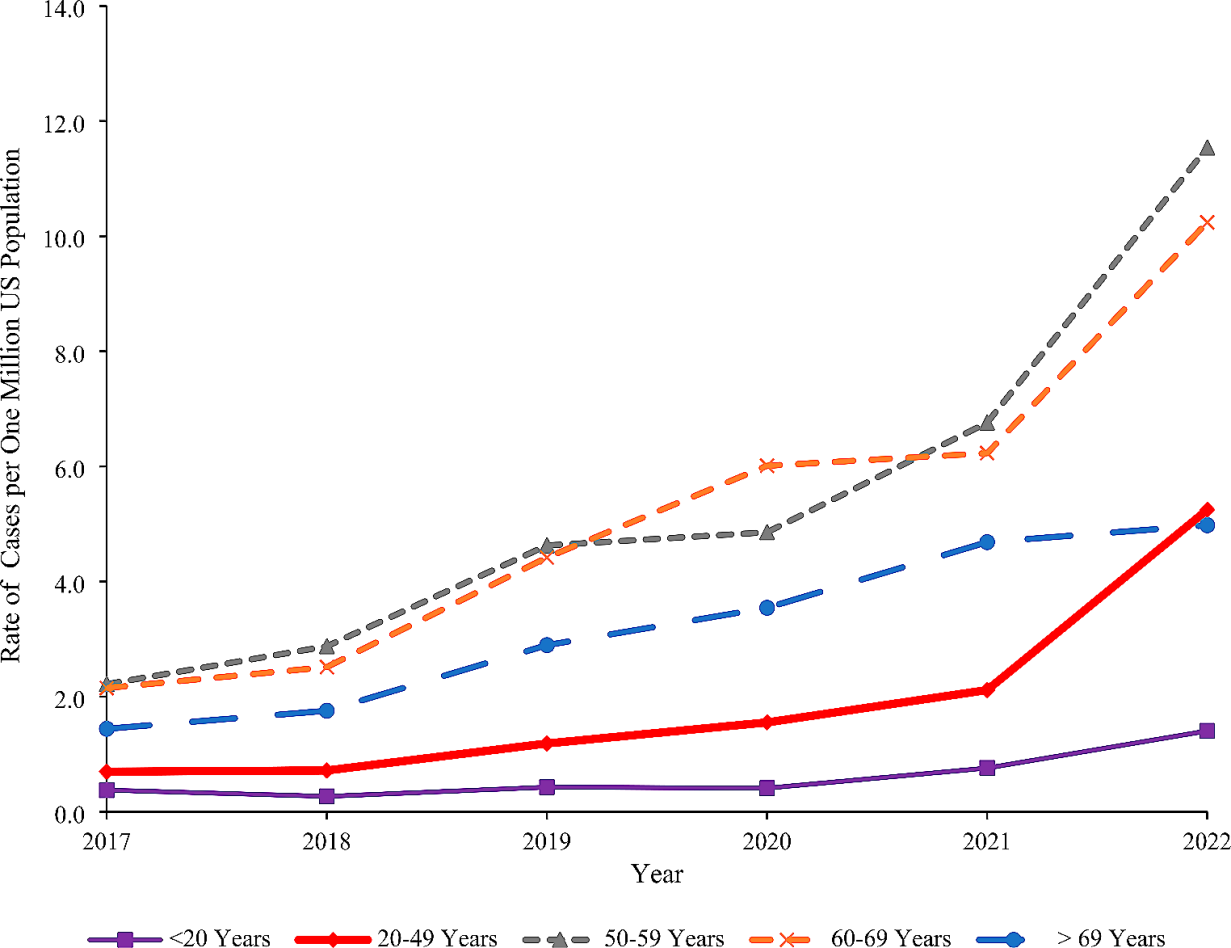
Rate of cases involving GLP-1 receptor agonists per one million US population reported to the NPDS by age group, 2017–2022

**Fig. 2:**
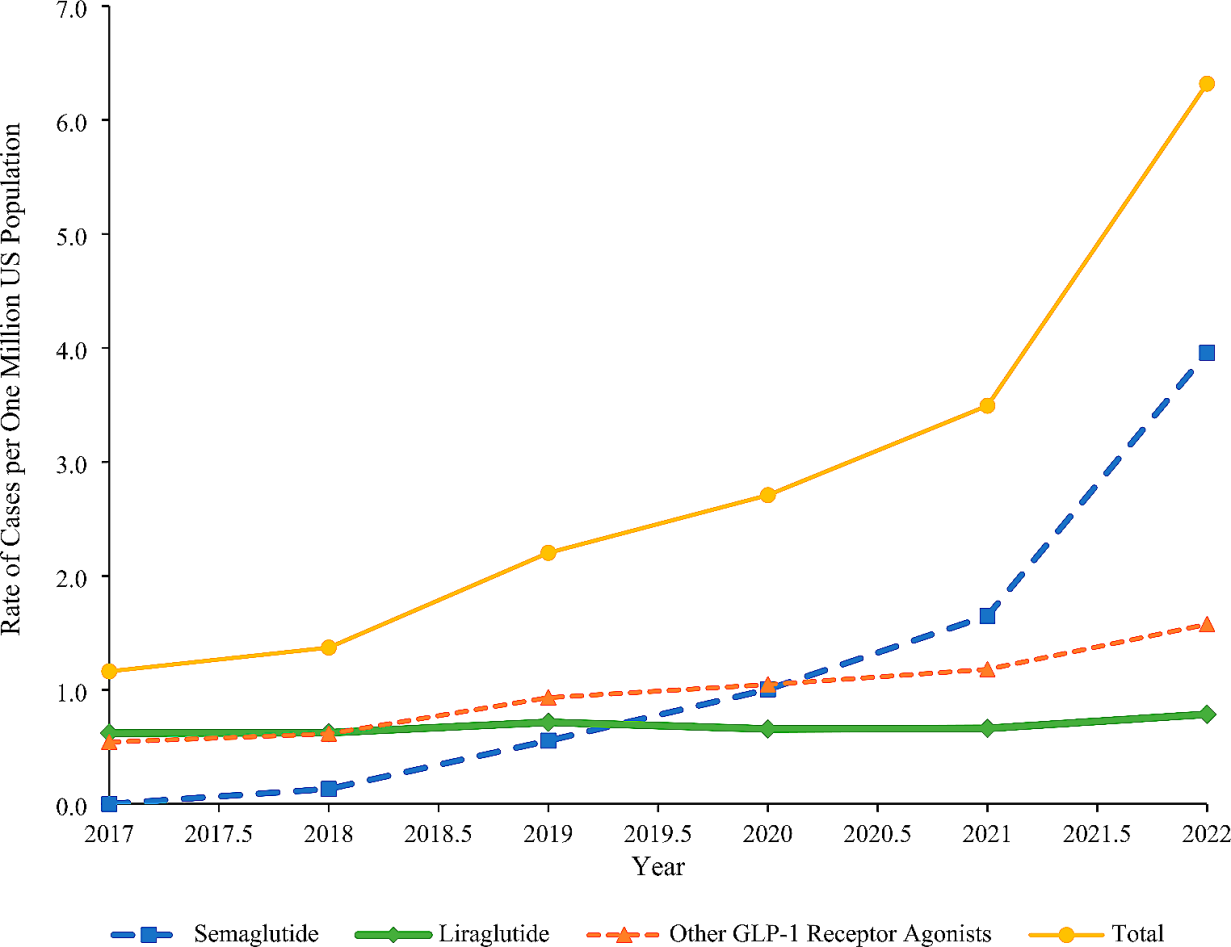
Rate of cases involving GLP-1 receptor agonists per one million US population reported to the NPDS by product group, 2017–2022

The rate of cases per one million US population increased from 1.16 in 2017 to 3.49 in 2021 and then rapidly increased by 80.9% to 6.32 in 2022. The increasing trend was observed across all age groups and both sexes; however, the rapid increase from 2021 to 2022 was not observed among < 20-year-olds, > 69-year-olds (Fig. 1), or males (Appendix 1). When product categories were examined separately, the rate per one million US population for semaglutide increased from 0.13 in 2018 to 1.65 in 2021 (no cases involving semaglutide were reported in 2017) and then rapidly increased by 139.8% to 3.96 in 2022. The rate for “other GLP-1RAs” showed a consistent increase from 0.54 in 2017 to 1.58 in 2022, while the rate for liraglutide remained relatively constant throughout the study period (Fig. 2).

**Fig. 3:**
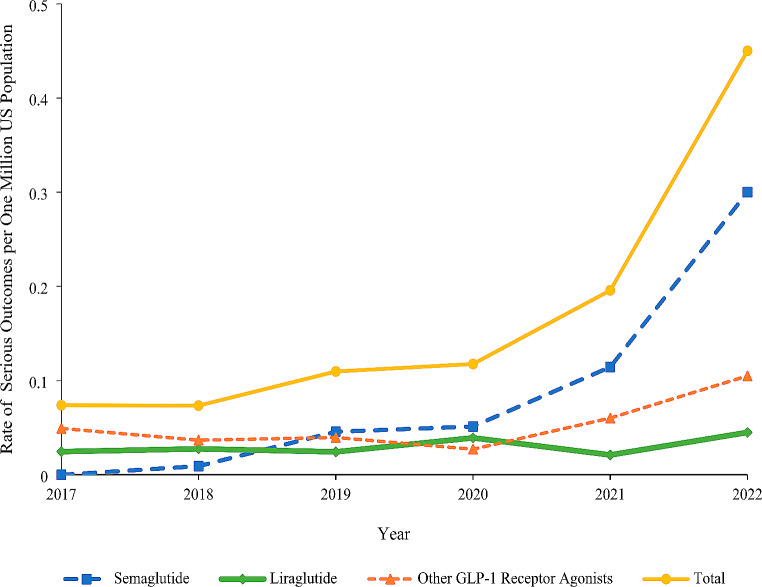
Rate of serious medical outcomes involving GLP-1 receptor agonists per one million US population reported to the NPDS by product group, 2017–2022

The rate of serious medical outcomes per one million US population increased from 0.07 in 2017 to 0.20 in 2021 and then rapidly increased by 129.9% to 0.45 in 2022 (Fig. 3). The upward trend was observed in all age groups and was primarily driven by increases in rates among 20-49-year-olds, 50-59-year-olds, and 60-69-year-olds (Appendix 2). When product categories were examined separately, the rate of serious medical outcomes for semaglutide increased from 0.01 in 2018 to 0.11 in 2021 and then by 162.2% to 0.30 in 2022; the rate of serious medical outcomes remained relatively constant for liraglutide and “other GLP-1RAs” (Fig. 3).

**Fig. 4:**
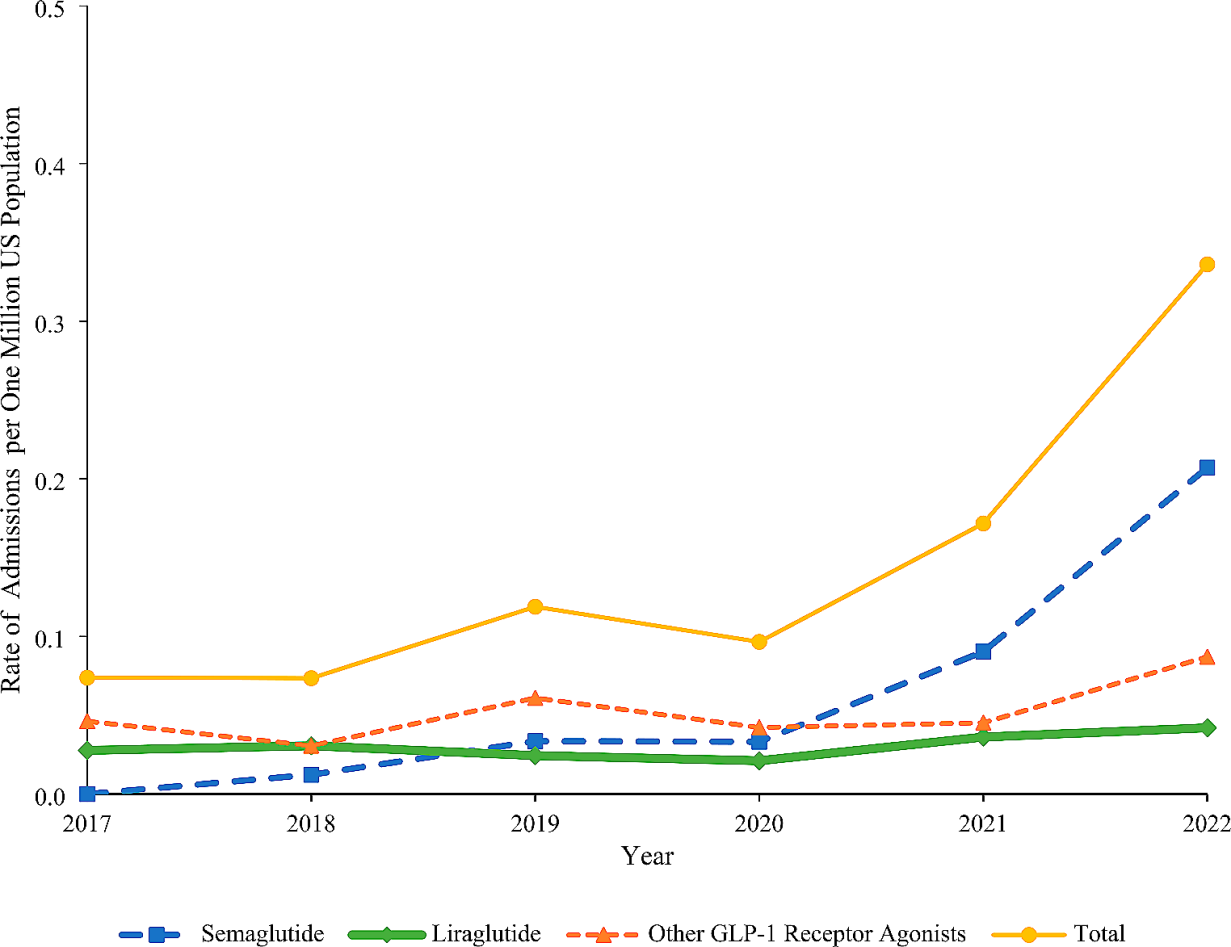
Rate of admission to a health care facility involving GLP-1 receptor agonists per one million US population reported to the NPDS by product group, 2017–2022

The rate of admission to a HCF per one million US population increased from 0.07 in 2017 to 0.17 in 2021 and then rapidly increased by 95.8% to 0.34 in 2022 (Fig. 4). The upward trend in the rate was observed among 50-59-year-olds and 60-69-year-olds, and the trend among 20-49-year-olds remained relatively constant from 2017 to 2021 and then rapidly increased from 2021 to 2022 (Appendix 3). When product categories were examined separately, the rate of admission to a HCF for semaglutide increased from 0.01 in 2018 to 0.09 in 2021 and then by 129.1% to 0.21 in 2022, while the admission rate for liraglutide and “other GLP-1RAs” remained relatively constant throughout the study period (Fig. 4).

## Discussion

In this investigation of six years of GLP-1RA cases reported to US PCs, most cases were associated with no or minimal effects and most did not require referral for medical treatment in a HCF. However, there were a notable proportion of cases that experienced serious medical outcomes (6.2%) or were admitted to a critical care unit or non-critical care unit (5.0%). Over the study period, the overall rates of GLP-1RA-related cases, and those for serious medical outcomes and admissions to a healthcare facility, increased. When examining specific product groups, semaglutide demonstrated greater accelerations in these rates over time than liraglutide and other GLP-1RAs, especially during the latter part of the study period. These increasing rates are likely reflective of the surge in GLP-1RA prescribing by clinicians in response to a combination of demonstrated clinical efficacy for management of diabetes and obesity [13, 16] as well as intense public interest. The larger increases in rates observed with semaglutide may be attributable to higher prescribing rates associated with the medication’s popularity [6], additional available formulations [29], and studies demonstrating clinical efficacy [30].

Although clinical trials have demonstrated the safety and tolerability of GLP-1RAs, side effects, especially gastrointestinal-related, are known to affect up to 10–40% of users [13, 15]. The most common related clinical effects reported for cases in our study were consistent with previous reports in the literature for this medication class [13–15, 17]. Adverse effects are more common during the early phase of treatment, when doses are initiated or increased [15, 31]. Adverse effects from GLP-1RAs can be mitigated through pausing up-titration or dose reduction [15, 32]. For patients experiencing profound side effects, medication discontinuation often leads to symptom abatement. Thus, most GLP-1RA side effects, especially gastrointestinal side effects such as nausea, vomiting, or diarrhea, can be managed successfully in the outpatient setting. Both providers and patients should be cognizant that serious side effects can occur and that even desirable effects of GLP-1RAs, such as early satiety, must be monitored closely, because they may affect nutrition or hydration status. In fact, a recent scientific abstract described a case of life-threatening starvation ketoacidosis preceded by 5 days of nausea and vomiting that developed several weeks after starting tirzepatide for weight loss [33]. Patients should be educated to recognize side effects so clinicians can address these promptly to prevent the development of complications. In addition, patients should be educated about the prevention of therapeutic errors, which accounted for more than three-fourths of reported cases. Dosing schedule systems and reminders may help prevent common scenarios, such as taking/giving the medication twice or too close together. Although reported for < 5% of cases in this study, providers should also be aware of the potential for patients to intentionally misuse/abuse GLP-1 agonists or use them for self-harm. This may be more common in populations with mental health or eating disorders, and screening for these conditions should be part of a clinician’s approach when considering this class of medications.

Certain adverse effects associated with GLP-1RAs, such as biliary disease [34, 35], acute kidney injury [36, 37], gastroparesis, or bowel obstruction [21] are more likely to require escalation of care. Additionally, pancreatitis is listed as a possible side effect on prescribing information of GLP-1RAs [38, 39], though this risk has not been clearly described in clinical trials [15, 40]. In our study, we identified only one case with elevated AST, ALT > 1,000 (which could represent hepatobiliary disease), one case with pancreatitis, one case with ileus, and a small number of cases with elevated creatinine (5 cases), including one case with kidney failure. Ongoing post-marketing surveillance and additional studies are needed to further elucidate associations between GLP-1RA use and specific serious, long-term, or delayed adverse effects. Reporting side effects to the FDA MedWatch program is one way providers and patients can help facilitate ongoing surveillance efforts.

Based on case review, the fatality reported in the NPDS appears to be consistent with complications of stercoral colitis, a rare condition that occurs when constipation and fecal impaction progresses to colonic distension and inflammation. In stercoral colitis, dehydrated fecal matter (i.e., fecalomas) can apply pressure to the bowel wall mucosa, causing ulceration, ischemia, necrosis, or perforation [41, 42]. Prompt recognition and management are critical to avoid morbidity and mortality [41, 43]. To our knowledge, this condition has not been previously described in the literature as being associated with GLP-1RA use. Although most cases of GLP-1RA-associated constipation do not progress to stercoral colitis, providers should be aware of the potential risk of this condition.

The high demand for GLP-1RAs has precipitated international drug shortages [7, 44]. Consequently, some patients have turned to compounding pharmacies, which advertise compounded versions of the peptides, for easier and cheaper access to these medications. In the spring of 2023, the FDA issued a warning on compounded semaglutide, citing a lack of FDA approval, reports of adverse effects, and an unknown safety profile [45]. Concurrently, the Obesity Medicine Association issued a position statement reiterating the importance of a transparent drug supply with appropriate regulatory oversight [46]; in its concluding statements, the Obesity Medicine Association recommended that prescribers and patients avoid “compounded polypeptides from undisclosed sources” and that patients should be informed of “potential limitations of compounded peptides” [46, 47]. Further study and regulatory oversight are needed to ensure the safety and efficacy of the compounded peptide drug supply. In addition to concerns about the contents of compounded products and their safety, use of compounded semaglutide may place patients at greater risk of therapeutic errors. These products are offered in multidose vials, unlike the single-dose pen version of the FDA-approved product. Confusion about how to draw up the medication for self-injection and confusion about units (milliliters vs. milligrams vs. units) could lead to large — up to 10- or 20-fold — overdose of these products [48].

### Study Limitations

There are several limitations to this study. Because the NPDS documents voluntary, self-reported exposures, the PCs and America’s Poison Centers cannot completely verify the accuracy of the information. Not all GLP-1RA cases are reported to PCs; therefore, this study underestimates the number of these cases. Reporting bias may occur, for example, more serious cases may be more likely to be reported than milder ones. Exposure calls to PCs do not necessarily represent a true poisoning or overdose. Due to limitations in NPDS coding, we were unable to separate cases associated with compounded peptides versus FDA-approved medications. Additionally, the indication for which the individual was using a GLP-1RA (e.g., obesity, diabetes) cannot be determined from our data. The relationship of medication dose with related clinical effects, HCF admission, or medical outcome was not examined in this study. Data miscoding by PC personnel or product misidentification by callers may also affect the data presented. Analyses were limited to variables in the NPDS database and individual case notes were only obtained for the fatality in this study. Despite these limitations, the NPDS is a comprehensive, standardized national database commonly used for epidemiologic investigations of poisonings.

## Conclusions

Most cases involving a GLP-1RA reported to US PCs were associated with no or minimal effects and most did not require referral for medical treatment in a HCF. However, a notable minority of individuals experienced serious medical outcomes or admission to a healthcare facility. The rate of cases reported to US PCs increased during the study period, including an 80.9% increase from 2021 to 2022; in addition, serious medical outcomes and HCF admissions demonstrated similar large increases of 129.9% and 95.8%, respectively, from 2021 to 2022. These findings likely reflect increased prescribing by health care providers and the growing popularity of GLP-1RAs among the public. Opportunities exist to improve provider and patient education. Clinicians and patients should be aware of both the common and rare adverse effects of this medication class, so that side effects can be addressed promptly to prevent symptom progression and complications. Education about prevention strategies for common types of therapeutic errors is also important. The effects of long-term use of GLP-1RAs, especially those more recently introduced into the market, have not been completely elucidated and are an important area of future study because this class of medications is likely to be used by an increasingly greater population as they are studied for additional indications.

### Electronic Supplementary Material

Below is the link to the electronic supplementary material.

**Supplementary Material 1:Appendix 1.** Rate of cases involving GLP-1 receptor agonists per one million US population reported to the NPDS by sex, 2017–2022. **Appendix 2.** Rate of serious medical outcomes involving GLP-1 receptor agonists per one million US population reported to the NPDS by age group, 2017–2022. **Appendix 3.** Rate of admission to a health care facility involving GLP-1 receptor agonists per one million US population reported to the NPDS by age group, 2017–2022

## Data Availability

Data analyzed in this study were from the National Poison Data System, which is owned and managed by America’s Poison Centers. Data requests should be submitted to America’s Poison Centers.

## Abbreviations

CI: Confidence interval
FDA: United States Food and Drug Administration
GLP-1RA: glucagon-like peptide-1 receptor agonist
HCF: Healthcare facility
NPDS: National Poison Data System
OR: Odds ratio
PC: Poison Center
US: United States
