# Supplementary material for: Glucagon-Like Peptide-1 Receptor Agonist Cases Reported to United States Poison Centers, 2017–2022
Source: J Med Toxicol. 2024 Feb 29;20(2):193–204. doi: 10.1007/s13181-024-00999-x (PMC10959851; doi:10.1007/s13181-024-00999-x)
Supplement: Supplementary file 1 — Supplementary Material 1:Appendix 1. Rate of cases involving GLP-1 receptor agonists per one million US population reported to the NPDS by sex, 2017–2022. Appendix 2. Rate of serious medical outcomes involving GLP-1 receptor agonists per one million US population reported to the NPDS by age group, 2017–2022. Appendix 3. Rate of admission to a health care facility involving GLP-1 receptor agonists per one million US population reported to the NPDS by age group, 2017–2022 [file 13181_2024_999_MOESM1_ESM.docx]

**Appendix 1. Rate of cases involving GLP-1 receptor agonists per one million US population reported to the NPDS by sex, 2017-2022**

**Appendix 2. Rate of serious medical outcomes involving GLP-1 receptor agonists per one million US population reported to the NPDS by age group, 2017-2022**

**Appendix 3. Rate of admissions to a healthcare facility involving GLP-1 receptor agonists per one million US population reported to the NPDS by age group, 2017-2022**
